# Challenges in phytotherapy research

**DOI:** 10.3389/fphar.2023.1199516

**Published:** 2023-05-31

**Authors:** Alexander Panossian

**Affiliations:** ^1^ Phytomed AB, Västervik, Sweden; ^2^ EuroPharma USA Inc., Green Bay, WI, United States

**Keywords:** phytomedicine and ethnopharmacology, pleiotropic and selective action, multitarget therapy, network pharmacology, synergy and antagonism, reproducibility quality and efficacy, herbal medicines

## 1 Introduction

The exponentially increasing number of publications in phytomedicine and ethnopharmacology over the last decades indicates a growing interest in herbal medicines (Atanasov et al., 2021). These studies provide thorough information on the chemical composition of traditionally used herbal medicines and uncover molecular mechanisms of their pharmacological action. Meanwhile, the more is known, the more questions arise, e.g.,:

• What are the benefits and disadvantages of purified compounds compared to multi-component herbal preparations?
• Where is the borderline between selective and nonspecific pharmacological action of herbal medicines?
• Is it undoubtedly possible to expect the synergistic effect of two or more ingredients in their combination based on accrued knowledge of their traditional use or current progress in bioinformatics without experimental validation of predicted results?
• What are the benefits of implementing the network pharmacology and systems biology approaches into ethnopharmacological research?
• How to achieve reproducible therapeutic efficacy of herbal medicines?

This overview provides a scientific opinion on this topic and not pretending on a comprehensive review of the research in the field. That is out of the scope of the article, where only representative studies were selected in the discussion.

## 2 Discussion

### 2.1 Benefits and disadvantages of purified mono-drugs vs. multi-component herbal medicines. Selective and nonspecific pharmacological action of herbal preparations

The endless debates between proponents of reductionistic and wholistic concepts in drug discovery research should reach a compromise since both approaches have advantages and disadvantages (Van Regenmortel, 2004; Hopkins, 2008; Klipp et al., 2020; Panossian and Efferth, 2022),

Purified compounds are easier to control to ensure their reproducible quality and efficacy. On the other hand, complex herbal preparation can be more effective due to their multitarget effects on various governing mechanisms of the regulatory systems involved in the pathogenesis of disorders and progression of diseases (Panossian, 2017; Panossian et al., 2018a; Panossian et al., 2021a; Yang and Wang, 2021; Panossian and Efferth, 2022).

The search for a “magic bullet” selectively targeting a single receptor specifically responsive to a pharmacological action is far from reality for at least two reasons.

One is related to the pharmacophoric moieties of ligands (pharmacologically active plant secondary metabolites). They can interact with various degrees of affinity to the same receptor sites allocated at several parts of macromolecules involved in many regulatory processes (Gertsch, 2011).

**FIGURE 1:**
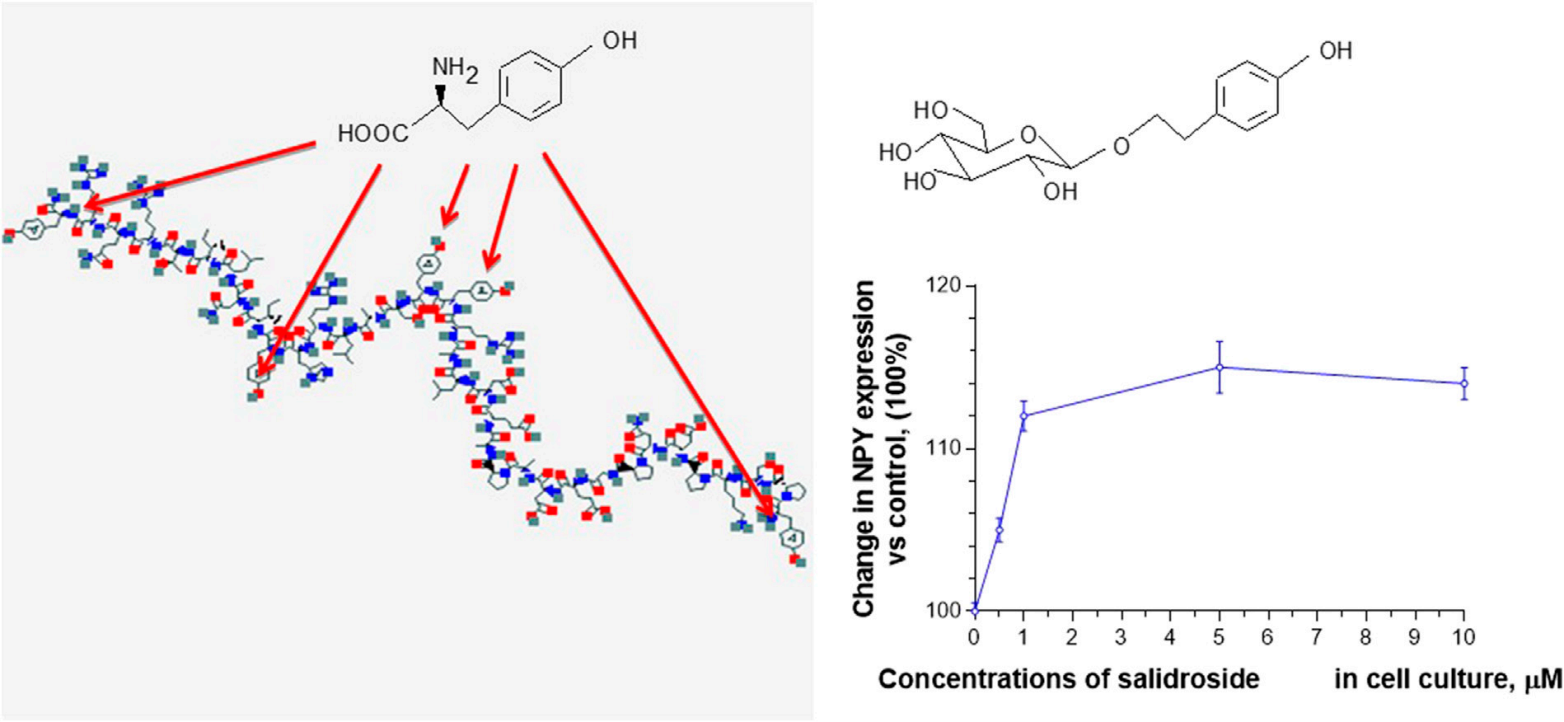
Effect of salidroside on expression of NPY in neuroglia, updated and adapted from Panossian et al. (2012) and from authors’ drawings.

For example, salidroside, which dose-dependently stimulates NPY-mediated expression and release of Hsp72 in human neuroglia cells (Panossian et al., 2012), has the same aromatic pharmacophore, the p-hydroxyl-methylene residue as tyrosine moiety, which is essential for brain receptor binding and NPY activity in many regulatory processes (Martel et al., 1990). We hypothesized that the p-hydroxyl-methylene residue of five tyrosine units (in the polypeptide chain of NPY) and the p-hydroxyl-ethylene residue of tyrosol and salidroside could compete on the NPY receptor binding site, Figure 1 (Panossian et al., 2012).

The pharmacological and toxicological outcomes of these interactions depend on the concentrations. At low concentrations, only a high affinity of the ligand-receptor infection may contribute to the comparatively “selective” effect. In contrast, in high concentrations, the interactions with other proteins may be toxic and less effective (Panossian et al., 2021a; Panossian et al., 2021b; Panossian et al., 2021c).

Furthermore, some plants’ secondary metabolites, such as toxoflavin, epigallocatechin gallate, genistein, and resveratrol, are known as so-called PAINS (pan assay interference compounds) and IMPS (invalid metabolic panaceas) *in vitro* studies (Baell and Walters, 2014), particularly in concentrations that cannot be achieved in humans due to their low bioavailability.

The second reason is that the pathogenesis and progression of diseases, as well as the recovery of the organisms, are multi-step processes where many players and regulatory systems are involved in the networks of dynamic interactions. These interactions result in various pharmacological effects and outcomes of functional systems.

Therefore, it is not surprising that many comprehensive reviews on the pharmacology of herbal medicines include the same type of activities, like immunomodulatory, anti-inflammatory, antioxidant, neuroprotective, hepatoprotective cardiovascular, vasoactive, etc., that are associated with the presence of the same classes of natural compounds, terpenoids, various phenolic compounds, etc. (Ahsan et al., 2020; Khan et al., 2021; Kumar et al., 2021; Ratan et al., 2021; Paul et al., 2021; Zeng et al., 2022).

### 2.2 Favorable and unfavorable outcomes of synergistic and antagonistic interaction components of multi-component herbal preparations

The use of complex formulations comprising fixed combinations of several plant extracts in TCM, Kampo, and other traditional medical systems suggests that they are more effective and presumably less harmful (in low dose) than their ingredients due to additive (1 + 1 = 2), potentiating (0 + 1 > 1), amplifying (1 + 1 >2) and synergistic (0 + 0>0) interactions of the components of complex herbal preparations. Hypothetically, antagonistic (1 + 1 < 2) and attenuating (1 + 1 < 2) interactions of the components of complex herbal preparations may also have an impact on the overall toxicity (Panossian et al., 2018b).

**FIGURE 2:**
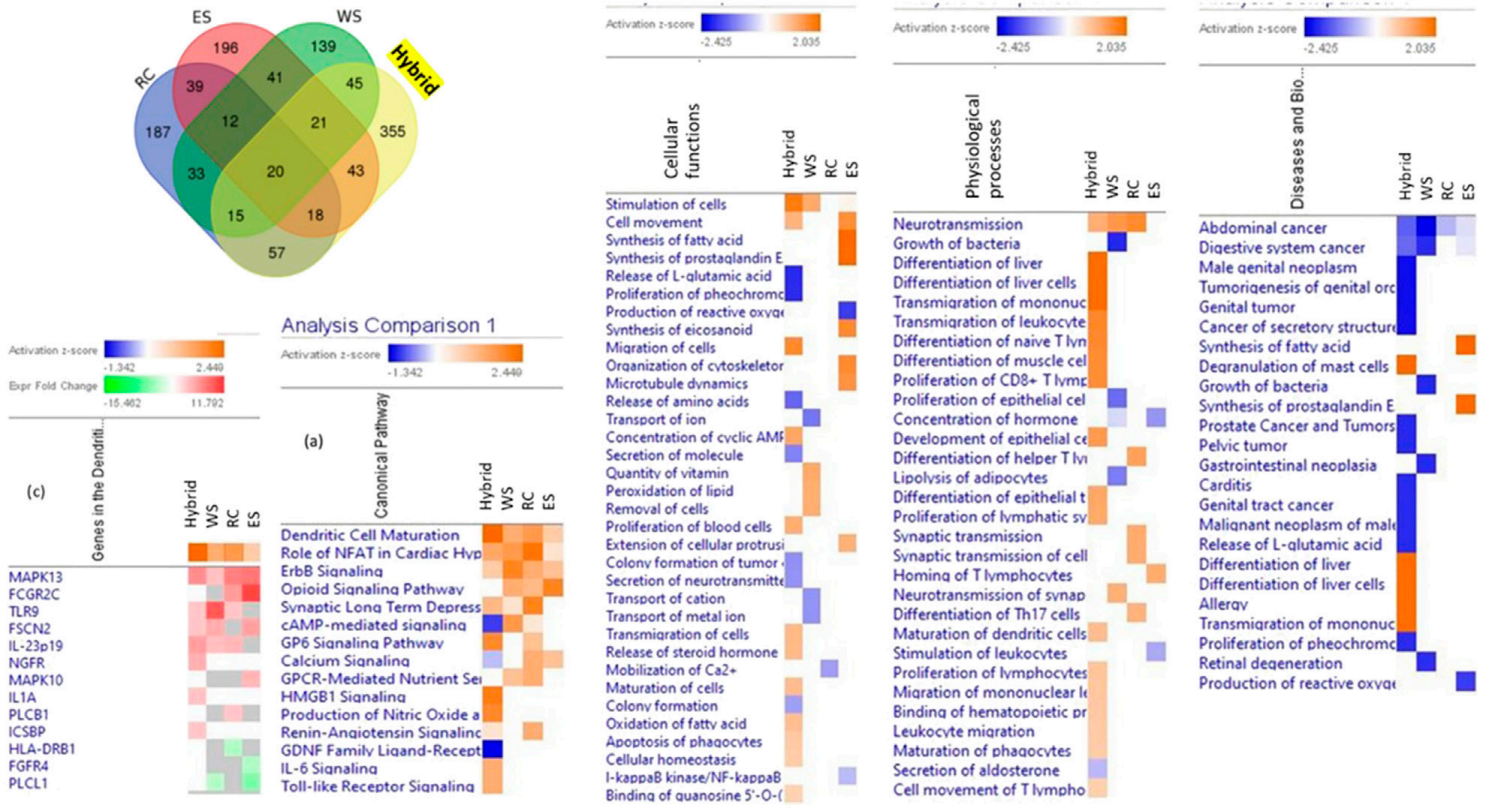
Venn diagrams of deregulated genes induced by treatment of neuroglial cells with *Rhaponticum cartamoides* L. (RC), *Eleutherococcus senticosus* (RS), and *Withania somnifera* (WS) root extracts and their hybrid combination (RC-ES-WS). Values indicate the number of unique genes up- or downregulated by each extract alone and the number of deregulated genes that overlapped multiple extracts. Heatmaps of canonical pathways, cellular functions, physiological processes, and diseases activated (brown) and inhibited (blue) by treatment of neuroglial cells with WS, RC, ES and the hybrid combination RC-ES-WS. Synergistic or antagonistic effects on canonical pathways, cellular functions, physiological processes, diseases, and gene expression associated with the pathway (e.g., dendritic cell maturation) can be observed by comparison of the effects of the hybrid substance RC-ES-WS with a lack of (or opposite) effects of individual extracts at a significance level of *p* < 0.05 (-log = 1.3) and a z-score >2. Upregulated genes are shown in red color, while and downregulated genes–in green color, updated and adapted from Panossian et al. (2018b) and from authors’ drawings.

These assumptions were supported in the series of *in vitro* studies where the effects of several plant extracts and their hybrid combinations on the number and composition of deregulated genes in brain cell cultures were analyzed (Panossian et al., 2013; Panossian et al., 2015; Panossian et al., 2018a; Panossian et al., 2018b). The composition of genes deregulated by hybrid combinations of plant extract was quantitatively and qualitatively different from the composition of genes deregulated by each plant separately, suggesting that the impact of the hybrid combination on the target cells was qualitatively different of the effects of ingredients, Figure 2 (Panossian et al., 2018a). In other words, the phytochemicals of two of 3 plant extracts exhibit quite different pharmacological activities when combined (Panossian et al., 2013; Panossian et al., 2015; Panossian et al., 2018a; Panossian et al., 2018b). These findings are essential for understanding unpredictable results obtained in clinical studies of multi-component drugs and dietary supplements (Jenkins et al., 2018; Boyle, et al., 2022a; Boyle, et al., 2022b; Noah et al., 2022).

An amazing example of the synergistic action of two plants is the hallucinogenic beverage Hoasca, used as the “vine of the soul” in religious cults of Indians living in the Amazon area (Barbosa et al., 2018; Santos et al., 2020). Hoasca/Ayahuasca is the decoction made from the bush *Psychotria viridis* Ruiz and Pav, which contains N, N-dimethyltryptamine (DMT), and the liana *Banisteriopsis caapi* (Spruce ex Griseb.) C.V Morton contains the β -carboline alkaloids harmine, harmaline, and tetrahydroharmine (THH) (Barbosa et al., 2018; Santos et al., 2020; Callaway et al., 1999; Barbosa et al., 2016). DMT has a potent, short-acting, hallucinogenic effect when smoked or used intravenously due to its actions on the brain serotonin receptors 5-HT2, 5-HT1a, and 5-HT-protein transporter (Callaway et al., 1999; Barbosa et al., 2016). Oral intake of DMT is ineffective due to metabolic oxidation of DMT by intestinal MAO, but it can be reversed when intestinal MAO is inhibited, as it actually happens on drinking Hoasca (Callaway et al., 1999; Barbosa et al., 2016). The psychic effects of Hoasca result from the inactivation of MAO present in the intestines, thus protecting DMT from oxidative deamination and enabling it to reach the brain through the bloodstream (Callaway et al., 1999; Barbosa et al., 2016).

Based on the assumption of synergistic interaction of several components, researchers propose that combinations of several active ingredients in one formulation can have superior effectiveness and better efficacy due to multiple effects on various targets (Efferth and Koch, 2011; Panossian et al., 2013; Panossian et al., 2015; Panossian et al., 2018b).

However, these expectations and predictions based exclusively on *in silico* analysis might be wrong due to unknown interactions and crosstalk between molecular targets within molecular networks involved in the cellular and overall response of organisms on the intervention of plants’ secondary metabolites in pathologic conditions. Several studies in humans have not found combined supplementation to be useful for preventing diseases and decreasing mortality (Watkins et al., 2000; Park et al., 2011). The systematic reviews and meta-analyses of randomized controlled trials of Supplemental Vitamins and Minerals on cardiovascular disease outcomes and all-cause mortality showed generally moderate- or low-quality evidence for preventive benefits (folic acid for total cardiovascular disease, folic acid and B-vitamins for stroke), no effect (multivitamins, vitamins C, D, β-carotene, calcium, and selenium), or increased risk (antioxidant mixtures and niacin for all-cause mortality) [Jenkins et al. , 2018]. The results of these studies led to the “Poisoned Chalice” hypothesis, suggesting that excessive multi-supplementation could interfere with molecular crosstalk involved in the regulation of the maintenance of cellular and systems homeostasis resulting in adverse physiological responses (Rutledge and Rose, 2015). Furthermore, the overall efficacy of treatment significantly depends on the diet backgrounds, e.g., the lifespan-extending effect of *Rhodiola rosea* extract in flies is dependent on dietary carbohydrate and caloric contents coupled with interaction with complex dietary components present in bananas, barley, or corn (Rutledge et al., 2021).

### 2.3 Incorporating the network pharmacology and systems biology approaches into ethnopharmacological research

Based on the ligand–receptor interaction, the reductionist concept is not an appropriate model for assessing the efficacy and safety of herbal preparations affecting multiple physiological functions, revealing polyvalent pharmacological activities, and are traditionally used in many conditions (Panossian and Efferth, 2022; Panossian et al., 2021a; Zhang et al., 2019; Noor et al., 2022; Li and Zhang, 2013; Han et al., 2019; Zhou et al., 2020; Jiao et al., 2021).

The network pharmacology approach is more suitable for understanding their mechanisms of action and predicting possible toxic effects, new indications for use, and lack of activity (Zhang et al., 2019; Noor et al., 2022; Li and Zhang, 2013; Han et al., 2019; Zhou et al., 2020; Jiao et al., 2021; Boezio et al., 2017; Chaudhari et al., 2020; Ma et al., 2021 Zhao et al., 2022; Cheng et al., 2018; Wang et al., 2021; Panossian and Efferth, 2022; Panossian et al., 2013; Panossian et al., 2018a; Panossian et al., 2021c).

However, the results of *in silico* analysis must be based on experimental findings of gene and protein expression of isolated cells (Ma et al., 2021 Zhao et al., 2022; Panossian and Efferth, 2022; Panossian et al., 2013; Panossian et al., 2018a; Panossian et al., 2021c) and validated at least *in vivo* experiments on rodents followed by clinical studies in humans (Cheng et al., 2018; Wang et al., 2021).

### 2.4 Reproducibility and consistency of the results of pharmacological and clinical studies of therapeutic efficacy of herbal preparations

Typically, plant extracts contain at least several active compounds comprising an active pharmaceutical ingredient/substance of herbal medicines, making it challenging to ensure their reproducible quality. Therefore, it is not surprising that systematic assessment and meta-analysis of pharmacological activity and therapeutic efficacy of herbal preparations often conclude that:

“Research regarding efficacy is contradictory” due to “a lack of independent replications in different studies. More research seems warranted, and rigorously-designed well-reported RCT that minimizes bias is needed” (Hung et al., 2011; Ishaque et al., 2012). Inconsistency of the results and lack of reproducible therapeutic efficacy of the same plant extracts in various studies is caused by many reasons, but primarily due to significant differences in the chemical composition of herbal preparations used in various studies. For example, chemical analysis of *R. rosea* L. roots and rhizome collected at different harvesting seasons from various geographic regions showed significant variability in the chemical composition and the amount of pharmacologically active compounds (Wiedenfeld et al., 2007; Dimpfel et al., 2018). A high degree of variation in the content of all active markers was observed in herbal extracts (Dimpfel et al., 2018) and Rhodiola products available to European buyers via the internet and other sources (Booker et al., 2016). The authors conclude that standardized content of active markers is necessary for the quality control of herbal preparations containing *R. rosea* extracts but insufficient for assessment of their potential efficacy; additional bioassays are needed to ensure the reproducible pharmacological activity of *R. rosea* extracts (Dimpfel et al., 2018). The same challenge exists for almost all herbal preparations on the world market.

Overall, “Good quality systems and manufacturing practices enable consumers to have confidence that products are authentic and meet a high specification for quality and safety (Booker et al., 2016; Heinrich et al., 2022; Durazzo et al., 2022; Heinrich et al., 2018).

The reproducible quality of herbal interventions is the primary issue in ensuring the reproducible efficacy of herbal medicines. Though, along with a lack of independent replications problem there are other concerns related to poor reporting and methodological quality issues, unclear risk of bias, optimized doses, and treatment regimens to rectify and observe the reproducible efficacy in various clinical studies (Kim et al., 2013; Lee et al., 2016; Li et al., 2021; Crawford et al., 2021; Wang et al., 2014; Liang et al., 2021). There is no clinical evidence that a steady-state concentration of active compound has been achieved during the treatment course. That is important to attain reproducible therapeutic efficacy since the concentration of active compounds in blood, and organ tissues significantly varies over time after drug administration from the baseline (0) to the maximal (Zhou et al., 2017). That might be crucial in some cases when dose-dependent reversal effects were observed (Panossian et al., 2021d).

## 3 Conclusion

This expert opinion and conclusions above are based on convincing observations suggesting further extensive research in the field.
